# The Association Between Tuberculosis and Osteoporosis: A Systematic Review and Meta-Analysis

**DOI:** 10.7759/cureus.76397

**Published:** 2024-12-26

**Authors:** Kridh Charatcharoenwitthaya, Kajorn Suntrapiwat, Wasit Wongtrakul

**Affiliations:** 1 International Demonstration School, Mahidol University, Nakhon Pathom, THA; 2 Pulmonary Diseases and Critical Care Medicine, Buddhachinaraj Hospital, Phitsanulok, THA; 3 Internal Medicine, Faculty of Medicine Siriraj Hospital, Mahidol University, Bangkok, THA

**Keywords:** epidemiology, fractures, meta-analysis, osteoporosis, tuberculosis

## Abstract

Recent research suggests that tuberculosis (TB) may pose a potential risk factor for osteoporosis, although the available evidence remains limited. This study aimed to comprehensively assess osteoporosis risk in TB patients through systematic review and meta-analysis methodology. Two investigators independently conducted a literature search using the Medical Literature Analysis and Retrieval System Online (MEDLINE) and Excerpta Medica Database (EMBASE) databases up to April 2024. Eligible longitudinal cohort studies had to evaluate the impact of active or a history of TB on the risk of osteoporosis and/or osteoporotic fractures. Point estimates and standard errors from each eligible study were pooled using DerSimonian and Laird's generic inverse variance method. Of 2,062 articles (1,765 from EMBASE and 297 from MEDLINE) reviewed, three retrospective cohort studies, comprising a total of 531,624 participants (174,726 patients with TB and 356,898 participants without TB), met the eligibility criteria and were included in the meta-analysis. The pooled analysis of three studies revealed an increased risk of osteoporosis among TB patients, with a pooled hazard ratio of 1.40 (95% CI, 1.26 - 1.57; *I^2^*= 54%). The pooled analysis indicated that populations with TB also had a higher risk of osteoporotic fractures than populations without TB, with a pooled hazard ratio of 1.65 (95% CI, 1.26 - 2.15; *I^2^*= 71%). Our systematic review and meta-analysis demonstrated a significantly increased risk of osteoporosis and osteoporotic fractures in patients with TB.

## Introduction and background

Tuberculosis (TB) is a chronic, communicable infectious disease caused by air-borne transmission of *Mycobacterium tuberculosis* [1]. Approximately 2 billion people worldwide have latent TB infection [1]. The pathogenesis of TB involves a complex host cell-mediated immune response to limit the proliferation of the mycobacterium [2]. About 5-10% of individuals with latent TB infection fail to adequately control the proliferation of mycobacteria, leading to the development of active TB, such as pulmonary TB, during their lifetime [1]. Standard treatments for pulmonary TB include a two-month intensive phase with anti-TB drugs (isoniazid, rifampicin, pyrazinamide, and ethambutol) followed by a four-month maintenance phase with isoniazid and rifampicin [3].

Osteoporosis, the most common metabolic bone abnormality, is characterized by diminished bone mass, connectivity, and structural integrity, resulting in skeletal fragility and an increased risk of fracture [4]. Established risk factors for osteoporosis include advanced age, female sex, low body mass index, calcium and vitamin D deficiencies, a sedentary lifestyle, and chronic inflammatory diseases [5]. Chronic infections can also contribute to systemic chronic inflammation, suggesting that chronic infections, including TB, might increase the risk of osteoporosis [6]. A recent cohort study from Taiwan reported an increased risk of osteoporosis and osteoporotic fractures in patients with TB [7]. However, evidence supporting this association is limited. Therefore, this systematic review and meta-analysis aimed to determine whether TB patients have a higher risk of osteoporosis and fractures compared to the general population by pooling data from available cohort studies.

## Review

Methods

Information Sources and Search Strategy

K.C. and K.S. independently conducted a systematic review of the literature in the Excerpta Medica Database (EMBASE) and Medical Literature Analysis and Retrieval System Online (MEDLINE) databases from their inception to April 2024 to identify all published longitudinal cohort studies examining the risk of osteoporosis and/or osteoporotic fractures in individuals with active or a history of TB. The search strategy, including terms for “tuberculosis” and “osteoporosis” is available as Supplementary Data 1. To ensure comprehensive identification of eligible studies, the review also included bibliographies of initially identified studies from EMBASE and MEDLINE. This study adhered to the guidelines outlined in the Preferred Reporting Items for Systematic Reviews and Meta-Analyses (PRISMA) statement. Only full-text articles in English were eligible for this meta-analysis. The study is registered on Open Science Framework, number osf.io/4ehd5.

Selection Criteria

Eligible studies were longitudinal cohort studies that assessed the impact of active or past TB on the risk of osteoporosis and/or osteoporotic fractures. Eligible cohort studies should present a hazard ratio (HR) with 95% confidence intervals (CI), comparing the incidence of osteoporosis and/or osteoporotic fractures between patients with and without TB. Any definitions of active or past TB, osteoporosis, and osteoporotic fractures used by the primary studies were accepted. Two researchers (K.C. and K.S.) independently assessed the eligibility of studies. Title and abstract reviews were performed to exclude studies that clearly did not fulfill the eligibility criteria. Then, the full-text review ensured that the included studies met all the eligibility criteria. Disagreements regarding eligibility were resolved through consultation with an experienced investigator (W.W.).

Data Extraction

The following information was extracted: the first author’s last name, year of publication, study design, country of study, number of participants, participant recruitment methods, methods for identifying and verifying the diagnosis of tuberculosis, osteoporosis and osteoporotic fractures, baseline participant characteristics, average follow-up duration (for cohort study), confounders adjusted in multivariate analysis, and adjusted effect estimates with corresponding 95% CI. The quality of the eligible cohort studies was independently appraised by two researchers (K.C. and K.S.) using the Newcastle-Ottawa Quality Assessment Scale [8]. Disagreements in scoring were settled through consultation with the experienced investigator (W.W.). 

Statistical Analysis

Review Manager 5.3 software (Cochrane Collaboration, London, United Kingdom) was used for all data analyses. DerSimonian and Laird's generic inverse variance method with a random-effects model was employed to pool point estimates from all included studies to estimate the pooled risk ratio. The weight of each study in the pooled analysis was inversely proportional to the magnitude of its standard error [9]. Cochran’s Q test with *I^2^* statistic was used to assess statistical heterogeneity. *I^2^* values between 0% and 25% suggested insignificant heterogeneity, 26% to 50% low heterogeneity, 51% to 75% moderate heterogeneity, and 76% to 100% high heterogeneity [10]. A funnel plot was visualized to evaluate publication bias.

Results

**Figure 1:**
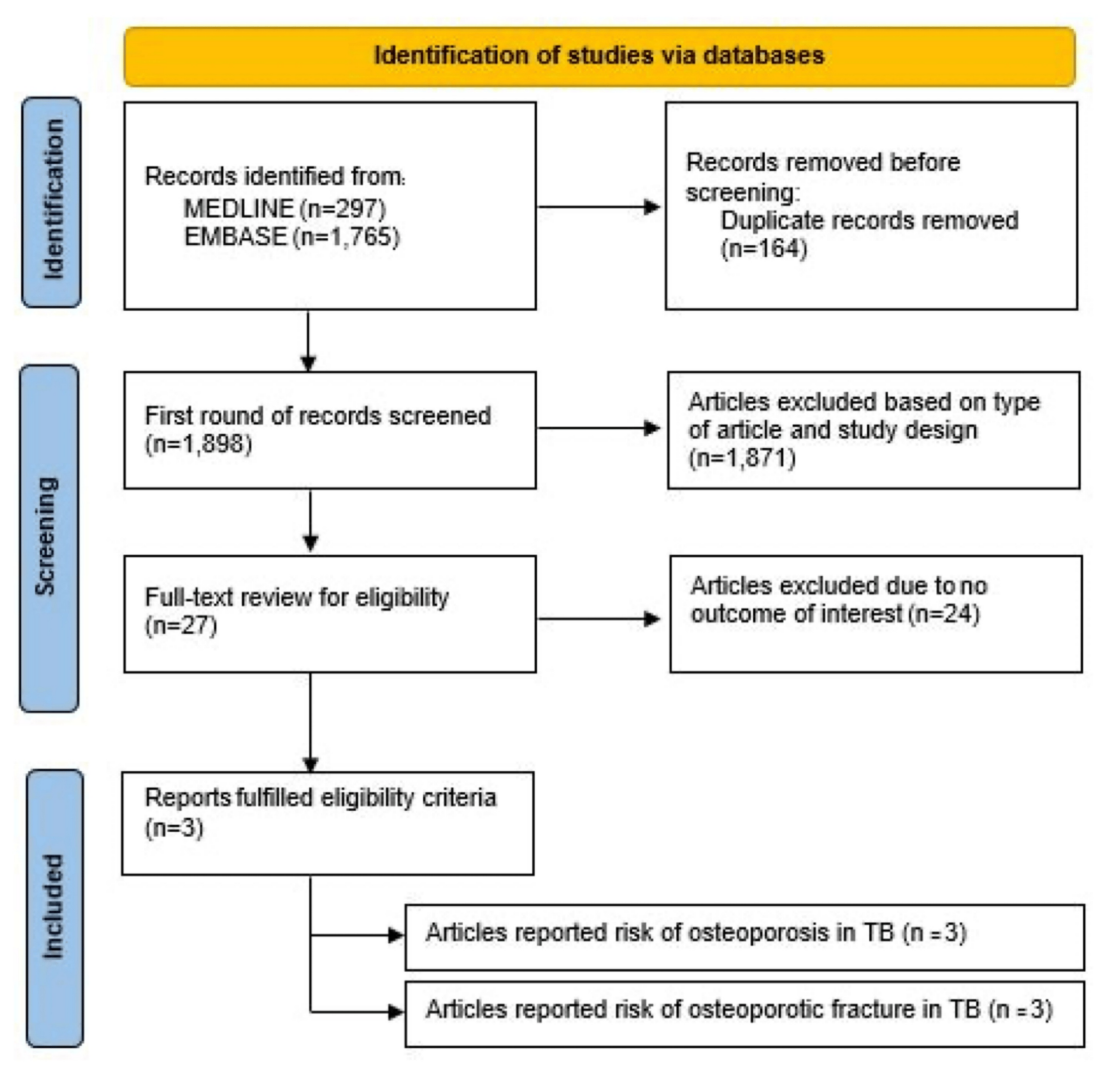
Flowchart of the literature review and study selection process

A total of 2,062 articles (1,765 from EMBASE and 297 from MEDLINE) were initially identified, with 164 duplicates subsequently removed, leaving 1,898 articles for title and abstract review. During the first round of review, 1,871 articles that clearly could not meet the eligibility criteria based on article type and study design were excluded. Consequently, a full-text review was conducted on 27 articles, of which 24 were excluded for not examining the relevant association of interest. The examination of the bibliographies of eligible studies did not reveal any additional studies fulfilling the inclusion criteria. Finally, the meta-analysis incorporated three retrospective cohort studies [7,11,12], comprising 531,624 participants (174,726 patients with TB and 356,898 participants without TB). All three cohort studies [7,11,12] compared not only the risk of osteoporosis between patients with and without TB but also the correlation between TB and osteoporotic fractures. Two studies were from Taiwan and one from South Korea. All three studies relied on International Classification of Diseases (ICD) coding to diagnose tuberculosis and osteoporosis/osteoporotic fracture. All studies adjusted for multiple potential confounders in multivariable Cox proportional hazard analysis. All studies had high Newcastle-Ottawa scores, implying a low risk of bias. Figure 1 provides an overview of the literature review and the process of selecting studies. Table 1 details the characteristics of participants and Newcastle-Ottawa assessment scales of the included studies.

**Table 1:** Characteristics of eligible studies in this study TB: Tuberculosis; ICD: International Classification of Diseases.

	Chen et al. (2017) [12]	Park et al. (2023) [7]	Yeh et al. (2016) [11]
Country	Taiwan	South Korea	Taiwan
Study design	Retrospective cohort	Retrospective cohort	Retrospective cohort
Total number of participants	Patients with TB: 3,725 Comparators: 14,900	Patients with TB: 164,389 Comparators: 328,778	Patients with TB: 6,612 Comparators: 13,220
Recruitment of participants	Participants were recruited through the National Health Insurance Research Database (NHIRD) between 2000 and 2012. Patients with active TB were identified using ICD-9-CM codes for TB with prescriptions of at least two anti-TB drugs for at least 28 days. Participants without TB were randomly selected as age- and sex-matched controls at a 1:4 ratio from the NHIRD.	Participants with the age of at least 40 years were recruited through the National Health Insurance Service-National Health Information Database (NHIS-NHID) between 2006 and 2017. Patients with newly diagnosed TB were identified using ICD-10 codes for TB and prescriptions of TB drugs for at least 28 days. Participants without TB were randomly selected with a 1:2 ratio marching for age, sex, income levels, residence, and registration from the NHIS-NHID	Participants were recruited through the Taiwan longitudinal health insurance database (LHID)between 1999 and 2005. Patients with newly diagnosed TB were identified using ICD-9-CM codes for TB. Participants without TB were randomly selected as age- and sex-matched controls at a 1:2 ratio from the LHID.
Diagnosis of tuberculosis	ICD-9-CM codes for TB and prescriptions of at least two anti-TB drugs for at least 28 days, with confirmation by two pulmonologists after reviewing clinical data	ICD-10 codes for TB and prescriptions of TB drugs for at least 28 days	ICD-9-CM codes for TB
Diagnosis of osteoporosis and fracture	ICD-9-CM codes for osteoporosis and osteoporotic fractures	ICD-10 codes for osteoporosis and osteoporotic fractures	ICD-9-CM codes osteoporosis and osteoporotic fractures
Follow up	All participants were followed until December 31, 2012, or until the diagnosis of osteoporosis, osteoporotic fractures, death, or withdrawal from NHIRD.	All participants were followed until December 31, 2017, or until the diagnosis of osteoporosis, osteoporotic fractures, or death.	All participants were followed until December 31, 2011, or until the diagnosis of osteoporosis, or osteoporotic.
Mean/median follow-up duration (years)	TB group: 5.4 Comparators: 6.0	7	7.7
Average age of participants (years)	TB group: 61.2 Comparators: 58.6	N/A	N/A
Percentage of male	76.8%	71.9%	69.7%
Variable adjusted in multivariate analysis	Age, sex, income level, urbanization, and comorbidities	Age, sex, income level, residence, comorbidities, body mass index, blood pressure, fasting blood glucose, total cholesterol, liver function test, alcohol and smoking	age, sex, drug use and comorbidities
Newcastle-Ottowa score	Selection: 4 Comparability: 2 Outcome: 3	Selection: 4 Comparability: 2 Outcome: 3	Selection: 4 Comparability: 2 Outcome: 3

Risk of Osteoporosis Among Patients With Tuberculosis

**Figure 2:**
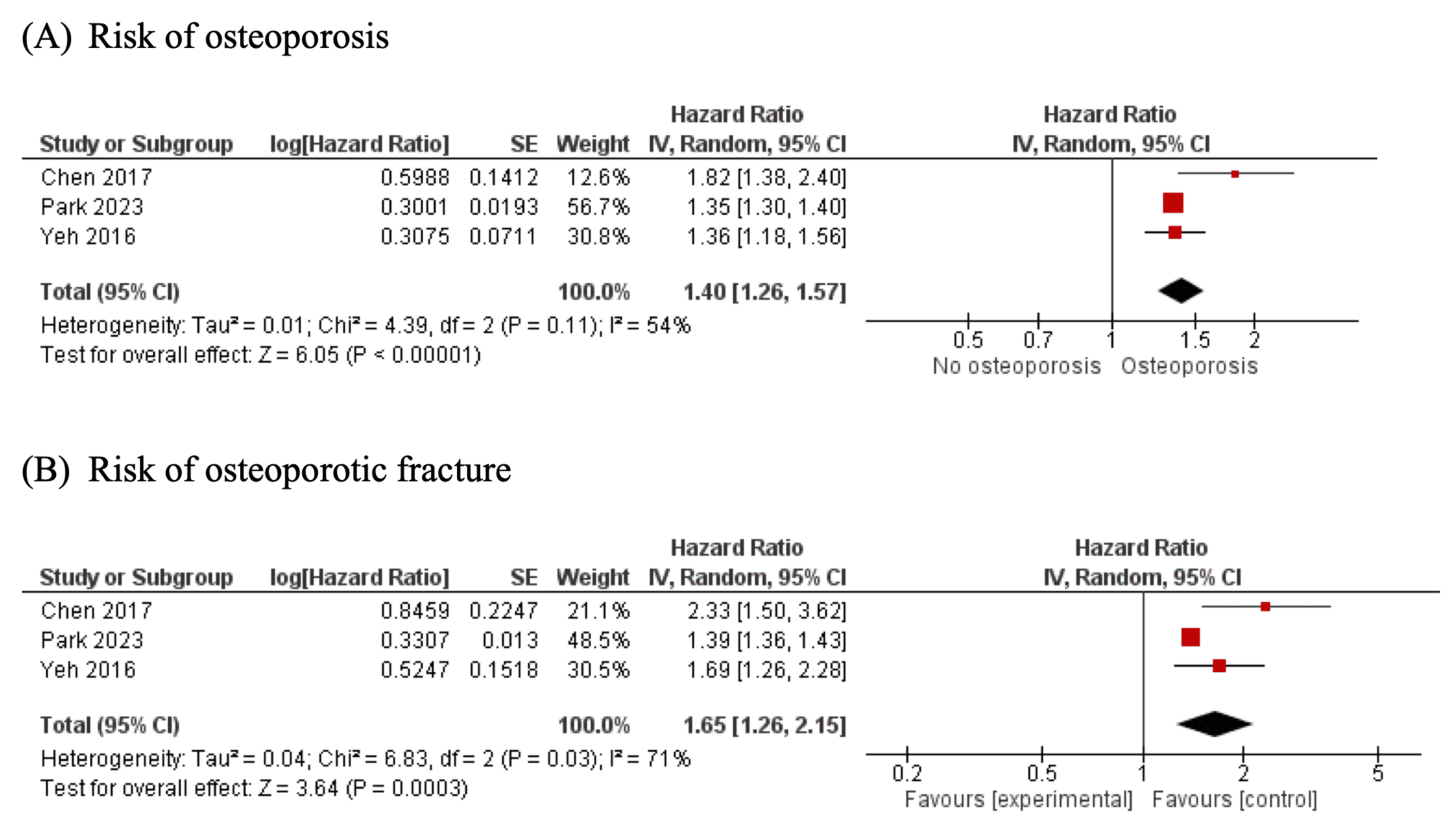
Forest plots from the meta-analysis The plot is showing the association between tuberculosis and (A) osteoporosis, and (B) osteoporotic fractures [7,11,12].

Figure 2A revealed a significantly increased risk of osteoporosis among tuberculosis patients compared to those without tuberculosis, with a pooled HR of 1.40 (95% CI, 1.26 - 1.57; *I^2^*= 54%). 

Risk of Osteoporotic Fracture Among Patients With Tuberculosis

Figure 2B reveals a significantly increased risk of osteoporotic fracture among tuberculosis patients compared to those without tuberculosis, with a pooled HR of 1.65 (95% CI, 1.26 - 2.15; *I^2^*= 71%).

Publication Bias

**Figure 3:**
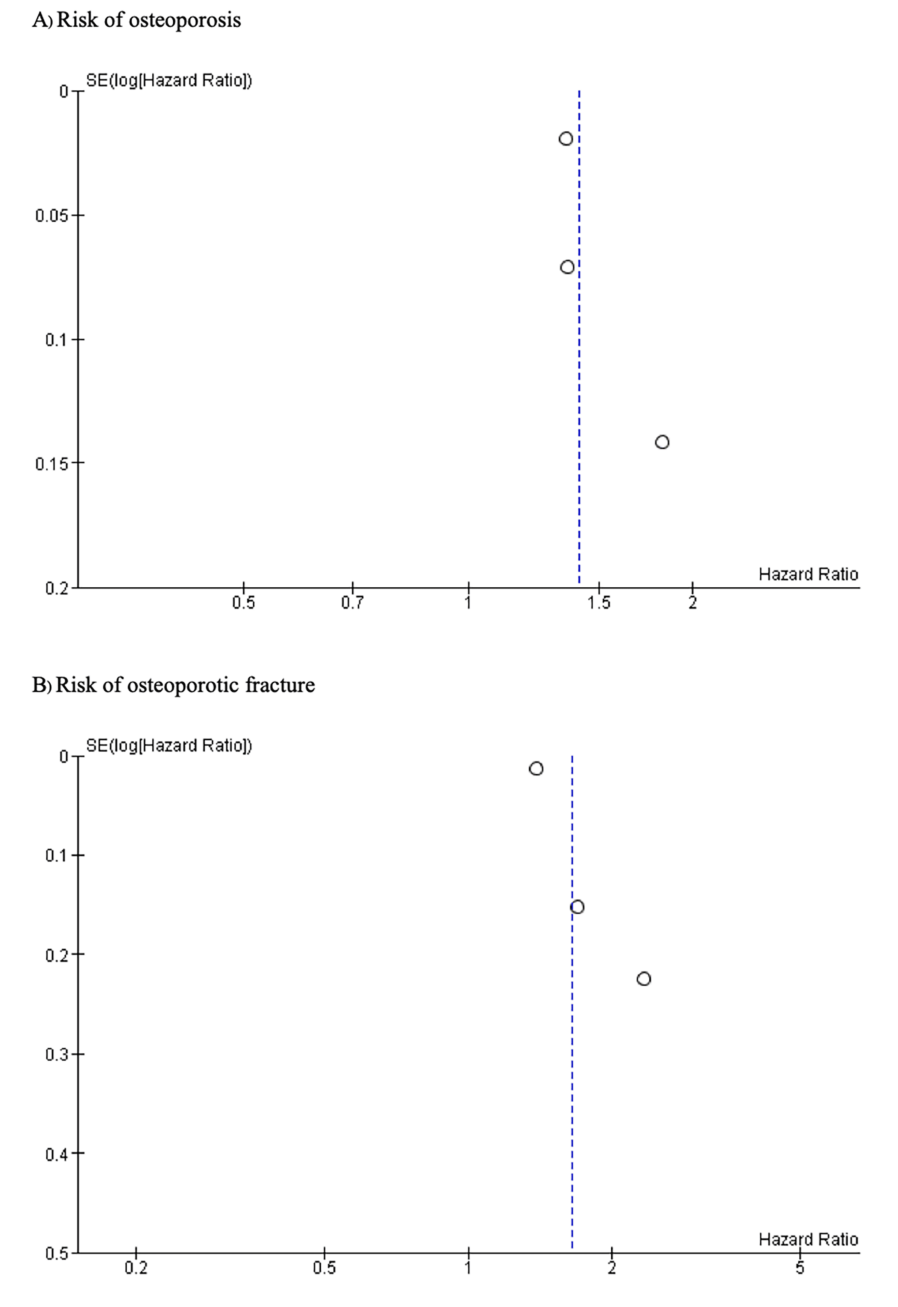
Funnel plots from the meta-analysis showing the association between tuberculosis and osteoporosis, and osteoporotic fractures (A) Osteoporosis, and (B) Osteoporotic fractures.

Visualization of funnel plots of both meta-analyses for the risk of osteoporosis and osteoporotic fracture in tuberculosis showed asymmetry, suggesting potential publication bias (Figures 3A, 3B).

Discussion

This study is the first systematic review and meta-analysis to comprehensively assess the risk of osteoporosis and/or osteoporotic fracture in patients with active or past TB compared to individuals without TB. The pooled analysis of early 531,624 participants found an approximately 1.4-fold increased risk of osteoporosis and a 1.6-fold increased risk of osteoporotic fracture among patients with TB. The suggested mechanisms for the observed relationship are discussed below.

One potential mechanism is chronic inflammation, a well-established risk factor for osteoporosis and osteoporotic fractures. Several chronic inflammatory diseases, such as rheumatoid arthritis [13], systematic lupus erythematosus [14], inflammatory bowel disease [15], and psoriasis [16], have been shown to be at significantly increased risk of osteoporosis and osteoporotic fractures in meta-analyses. Inflammatory conditions can increase bone resorption and diminish bone formation through elevated levels of pro-inflammatory cytokines [17]. Elevated levels of pro-inflammatory cytokines, such as tumor necrosis factor-α (TNF-α), interleukin (IL)-1, and IL-6, promoted resorptive action on osteoclasts through systemic activation and production of receptor activator of nuclear factor kappa-B ligand [17,18]. TNF-α also inhibited the differentiation of osteoblasts through nuclear factor kappa-B and mitogen-activated protein kinase pathways [19]. TB, a chronic infection, usually has a higher inflammatory burden. TB induced the host’s body to release inflammatory cytokines such as TNF-α and IL-1 [20]. Therefore, these inflammatory cytokines in patients with TB lead to increased osteoclastogenesis and diminished osteoblastic activity, resulting in accelerated bone resorption and osteoporosis. 

Another possible mechanism is vitamin D deficiency. A 2021 meta-analysis of 12 studies with approximately 3600 patients shows that patients with TB are much more likely to have vitamin D deficiency than controls, with a pooled OR of 3.23 (95% CI, 1.91-5.45) [21]. Additionally, patients with TB usually have a higher prevalence of comorbidities, especially chronic kidney disease [22]. Chronic kidney disease is a significant risk factor for vitamin D deficiency due to the reduced activity of renal 1-alpha hydroxylase for the activation of active vitamin D [23]. However, a study by Yeh et al. demonstrates that in multivariate analysis adjusted for vitamin D supplementation and comorbidities, including chronic kidney disease, TB was still a significant risk factor for the development of osteoporosis (aHR of 1.69; 95% CI, 1.26-2.28)and osteoporotic fractures (aHR of 1.42; 95% CI, 1.25-1.61) [11]. 

Medications for TB treatment, especially isoniazid, may play a role in the development of osteoporosis. Isoniazid is the main medication for the treatment of TB [24]. Isoniazid can induce vitamin B6 or pyridoxine deficiency by competitively inhibiting the action of pyridoxine [25]. Pyridoxine deficiency can impair the cross-linking of collagen during bone formation, leading to reduced bone mass and osteoporosis [26]. Therefore, isoniazid can lead to pyridoxine deficiency and osteoporosis.

Low body mass index, a well-established risk factor for osteoporosis, may serve as a key mechanism linking TB to an elevated risk of osteoporosis. Patients with TB often present with lower body mass index due to the increased metabolic demand and malnutrition associated with the disease [27]. A low body mass index reflects reduced body fat, which is essential for vitamin D storage and the synthesis of hormones, such as sex steroids, that regulate bone metabolism, thereby increasing the risk of osteoporosis [28].

Notably, despite osteoporosis being more common in females, approximately 70% of participants in the included studies were males. Male patients with TB might have a higher risk of osteoporosis compared to males without TB due to the infection’s potential negative impact on testosterone levels. A study from Egypt revealed that men with pulmonary TB exhibited significantly lower serum testosterone levels compared to those without TB [29]. Declines in serum testosterone contribute to osteoporosis by impairing bone formation through reduced activation of androgen receptors in osteoblasts [30]. These findings underscore the importance of recognizing gender-specific risk factors in the TB population.

Surveillance bias may contribute to the apparent increased risk of osteoporosis, as patients with TB receive more extensive medical attention and monitoring due to the extended duration of treatment and higher prevalence of comorbidities, potentially leading to a higher likelihood of screening with dual-energy X-ray absorptiometry and osteoporosis diagnosis.

This meta-analysis has several strengths, including extensive adjustment for potential confounders such as comorbidities, especially chronic kidney disease, between TB and osteoporosis in the majority of included studies. Therefore, we believe that the observed association between TB and osteoporosis truly exists and is not a result of potential confounders. Moreover, analysis of pooled HRs from cohort studies allows us to establish a causal relationship between TB and the risk of osteoporosis and related fractures.

This meta-analysis has certain limitations that should be acknowledged. First, all three studies included diagnostic codes, and registry data were utilized for diagnosing TB, osteoporosis, and osteoporotic fractures. This approach might have limited the accuracy of case identification. Second, all the studies were conducted in East Asia, limiting the generalizability of our findings to other geographic regions. Variations in genetic predisposition, environmental factors, healthcare systems, and TB prevalence across different populations may influence the association between TB and osteoporosis. Third, all included studies employed retrospective cohort designs, which are inherently prone to confounding and selection bias. While the included studies accounted for key potential confounders such as comorbidities, residual confounding from unmeasured variables-such as dietary habits and genetic predispositions-remains a concern and could have influenced the observed associations. Fourth, moderate heterogeneity was observed in the pooled results (I² = 54-71%), reflecting variability among the studies in terms of populations, study designs, and methodologies. Although such heterogeneity is not uncommon in meta-analyses, the small number of included studies limited our ability to perform meaningful subgroup or sensitivity analyses to identify its sources. Finally, evidence of publication bias, as indicated by funnel plot asymmetry, suggests that studies reporting a positive association between TB and osteoporosis or osteoporotic fractures may be overrepresented. While statistical tests like Egger’s regression are valuable for quantifying publication bias, the small sample size in this meta-analysis reduces the reliability of such methods. These limitations underscore the need for further well-designed studies to validate our findings and address potential biases.

The findings of this meta-analysis emphasize the importance of proactive measures to prevent osteoporosis in patients with TB, particularly those with established risk factors such as advanced age, female sex, corticosteroid use, smoking, or comorbidities. Recommendations include baseline bone mineral density screening at the time of TB diagnosis or treatment initiation, with a follow-up scan upon completion of TB therapy to evaluate bone health. For patients with significant baseline bone loss or ongoing risk factors, repeat bone density testing every two years may be warranted. Management should encompass more than routine calcium and vitamin D supplementation, extending to pharmacological treatments like bisphosphonates or denosumab for those with osteoporosis (a T-score of -2.5 or less) or high fracture risk. Lifestyle modifications, including weight-bearing and resistance exercises, smoking cessation, alcohol moderation, and nutritional support for malnourished individuals, are integral to a holistic osteoporosis prevention strategy.

## Conclusions

The present systematic review and meta-analysis reveals a significantly increased risk of osteoporosis and osteoporotic fractures in patients with TB. This may warrant early intervention and counseling to prevent the development of osteoporosis, such weight-bearing exercise, adequate intake of vitamin D and calcium, and early screening for osteoporosis in patients with TB.

## Appendices

Supplementary Data 1 Search strategy

EMBASE

1. ‘tuberculosis’/exp OR ‘tuberculosis’; 2. ‘mycobacterium’/exp OR ‘mycobacterium’; 3. 'osteoporosis'/exp OR 'osteoporosis'; 4. 'osteoporotic fracture'; 5. 'fragility fracture'/exp OR 'fragility fracture'; 6. 'osteopenia'/exp OR 'osteopenia'; 7. 'bone density'/exp OR 'bone density'; 8. 'bone mineral density'; 9. #1 OR #2; 10. #3 OR #4 OR #5 OR #6 OR #7 OR #8; 11. #9 AND #10

MEDLINE

1. tuberculosis.mp. or exp Tuberculosis/; 2. mycobacterium.mp. or exp Mycobacterium/or exp Mycobacterium Infections/; 3. exp Osteoporosis/ or osteoporosis.mp.; 4. osteoporotic fracture.mp. or exp Osteoporotic Fractures/; 5. fragility fracture.mp.; 6. bone mineral density.mp. or exp Bone Density/; 7. osteopenia.mp.; 8. 1 or 2; 9. 3 or 4 or 5 or 6 or 7; 10. 8 and 9
